# Molecular Pathways Controlling Autophagy in Pancreatic Cancer

**DOI:** 10.3389/fonc.2017.00028

**Published:** 2017-03-03

**Authors:** Maria New, Tim Van Acker, Jaclyn S. Long, Jun-ichi Sakamaki, Kevin M. Ryan, Sharon A. Tooze

**Affiliations:** ^1^Molecular Cell Biology of Autophagy Laboratory, The Francis Crick Institute, London, UK; ^2^Tumour Cell Death Laboratory, Cancer Research UK Beatson Institute, Glasgow, UK

**Keywords:** autophagy, pancreatic cancer, PDAC, metabolism, autophagy inhibition

## Abstract

Pancreatic ductal adenocarcinoma (PDAC) is one of the few cancer types where the 5-year survival rate shows no improvement.

Despite conflicting evidence, the majority of data points to an essential role for autophagy in PDAC growth and survival, in particular constitutively activated autophagy, can provide crucial fuel to PDAC tumor cells in their nutrient-deprived environment.

Autophagy, which is required for cell homeostasis, can both suppress and promote tumorigenesis and tumor survival in a context-dependent manner. Protein by protein, the mystery of how PDAC abuses the cell’s homeostasis system for its malignant growth has recently begun to be unraveled. In this review, we focus on how autophagy is responsible for growth and development of PDAC tumors and where autophagy and the mechanisms controlling it fit into PDAC metabolism. Understanding the range of pathways controlling autophagy and their interplay in PDAC could open the way for new therapeutic avenues.

## Introduction

Pancreatic cancer is a disease in which malignant cells originate in pancreatic tissue, leading to over 200,000 deaths per year worldwide—making pancreatic cancer the ninth leading cause of death from cancer (1). Eighty-five percent of pancreatic cancer cases are pancreatic ductal adenocarcinomas (PDACs), and there is currently no effective screening tool to detect early malignant or premalignant tumors. This makes PDAC one of the most deadly common cancers, as diagnosis is most likely to be at an advanced stage, with metastatic or locally advanced disease (2). Median patient survival is only 6–9 months (3) and only about 4% of patients live 5 years after diagnosis (4).

Defining features of PDAC include a high rate of *KRAS* activating mutations (>90%), a reprogramming of cellular metabolism, a hypervascular and hypoxic microenvironment, and susceptibility to both local invasion and metastasis (2). Therapeutic resistance of PDAC to radiotherapy, targeted agents, and chemotherapy means that new therapeutic avenues are urgently needed. One avenue would be to target the autophagic pathway as a number of studies have linked autophagy to PDAC survival and progression.

Macroautophagy (hereafter referred to as autophagy) is an evolutionarily conserved membrane-mediated process that delivers cytoplasmic constituents to lysosomes for degradation and component recycling. This complex process is mediated by at least 18 autophagy genes (Atg genes) in mammals (5). Upon autophagy induction triggered by cell stress, double membrane autophagosomes form and engulf cytosolic proteins and damaged organelles, either through a non-selective process or a selective receptor mediated autophagy, such as mitophagy (6). Autophagy initiation is controlled by the ULK kinase complex and the VPS34 phosphoinositol-3-phosphate (PtdIns3P)-kinase complex containing Beclin-1, which integrate stress signals from the mTOR complex 1 (mTORC1). When mTORC1 activity is inhibited, the ULK and the Beclin-1 complex translocate to the initiation site marked by ATG9 (7). The production of PtdIns3P by the Beclin-1 complex allows binding of WIPI2, recruitment of ATG12-5-16, and lipidation of the LC3/GABARAP family (8). Lipidated LC3 (LC3-II) is required for autophagosome formation, and detection of LC3-II by immunoblotting or immunofluorescence is the most established method of monitoring autophagy.

In normal conditions, autophagy is a homeostatic mechanism that serves to degrade damaged proteins and organelles that may diminish cellular fitness and integrity. The levels of autophagy can also be changed in response to a variety of intracellular and extracellular stresses, such as starvation, ER stress, hypoxia, oxidative stress, and pathogen invasion. The role of autophagy in cancer is complex with both tumor-survival and tumor-suppressive roles, which are dependent on tumor type, stage, and genetic lesions. Autophagy is thought to inhibit malignant transformation under normal conditions and is required for anticancer immunosurveillance (9). However, autophagy in cells which are already malignant frequently supports tumor progression and anticancer therapy resistance, by providing a means for cells to survive intracellular and extracellular stress (9).

Autophagy is tightly regulated starting from transcriptional activation to posttranslational protein modification (10), and the regulation of autophagy in PDAC is gradually becoming elucidated. Transcriptional control of autophagosome–lysosome function has been shown to drive PDAC metabolism (11), whereas starvation-induced vacuolar protein 1 (VMP1) expression in pancreatic acinar cells drives early autophagy through VMP1 association with the early autophagic structures on the ER membrane (12, 13). Autophagy inhibition or loss has been shown to lead to tumor regression in PDAC xenograft models and death in PDAC cell lines (14). Autophagy supports PDAC cell survival by a range of mechanisms, including autophagic secretion of alanine by pancreatic stellate cells (PSCs) for tumor metabolism (15) and prevention of ER stress (16). The well documented role of autophagy for survival of PDAC and the potential for therapy through autophagy modulation has been explored in PDAC cell lines, where autophagy blockage has been shown to reduce chemoresistance (14). In one study involving a small number of human patients, inhibition of autophagy did not show any significant therapeutic effect (17).

The focus of this review will be the role of autophagy in PDAC, a cancer type in which extensive evidence currently points to a dependence on autophagy for tumor growth, development, and metabolism (14, 18), although there are also studies highlighting autophagy-independent PDAC cell line and tumors (19, 20).

## Dual Role of Autophagy in Cancer

**Figure 1:**
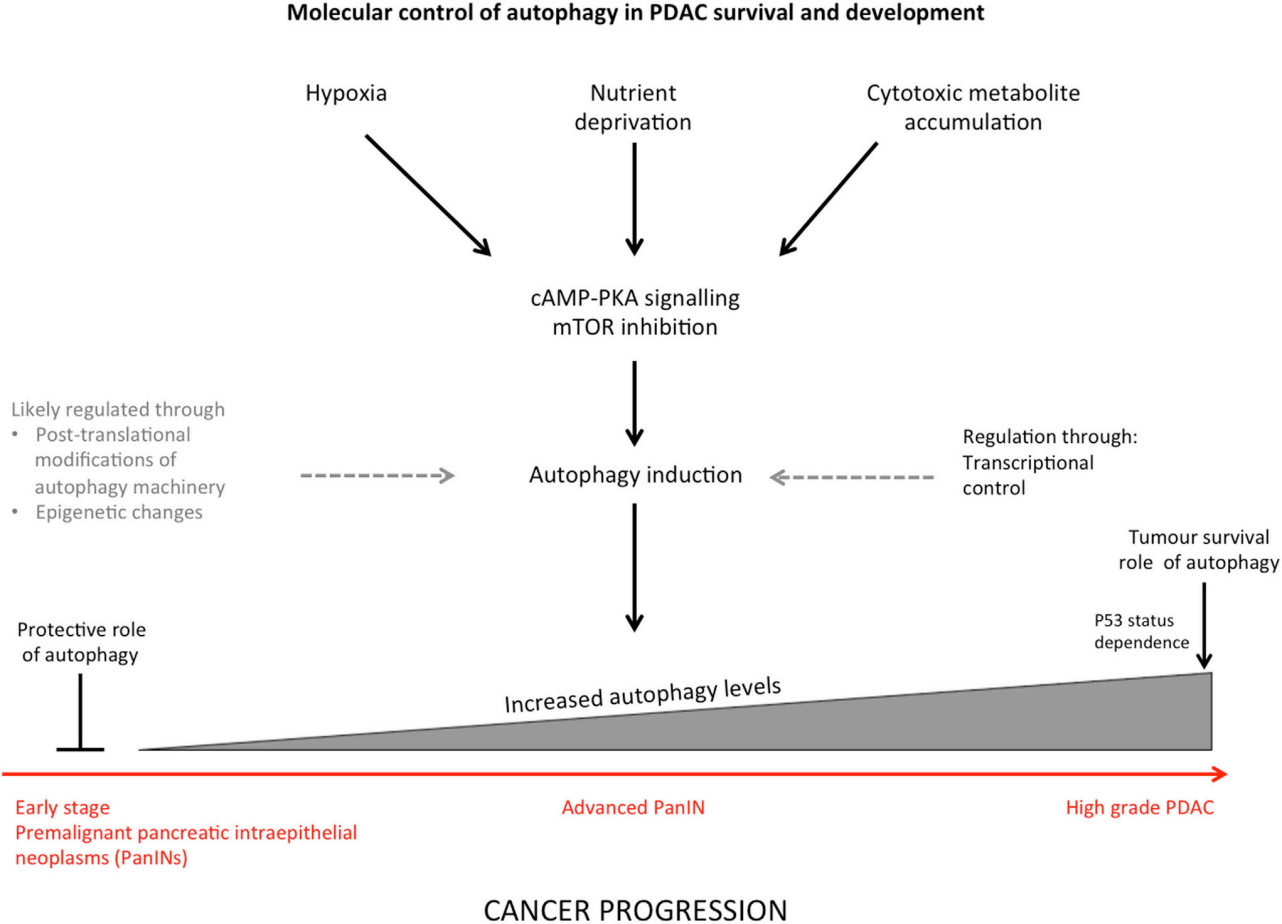
**Summary diagram indicating the stimuli that induce autophagy in pancreatic ductal adenocarcinoma (PDAC) and regulation of autophagy through different stages of cancer progression**.

It is now accepted that autophagy can suppress or promote tumorigenesis and tumor survival depending on cellular context and stage in tumor development, this characteristic is referred to as a “double-edged sword” (21) (Figure 1).

Defects in the autophagic machinery in mouse cancer models have been connected to malignant transformation in a number of studies, and indeed, the tumor-suppressive properties of Beclin-1 provided the first evidence of this (22). More recently, mice heterozygous for activating molecule in Beclin-1-regulated autophagy (AMBRA1) were also shown to have an increased rate of tumorigenesis (23). The scaffold protein AMBRA1 was shown to promote the binding of protein phosphatase 2A to the c-Myc transcription factor and, when mTOR is inhibited, causes c-Myc to be dephosphorylated consequently followed by a reduction in cell division (23). Other mechanisms through which autophagy functions in an oncosuppressive role include protection of the cell from mutagenic reactive oxygen species (ROS) accumulation, DNA damage, genomic instability, and oncogenic proteins, hyperactivation of which activates autophagy (9). While this suppressive function typically allows the cells to survive, prolonged autophagy activation may result in caspase-independent autophagic-programmed cell death (24). Autophagic cell death is poorly defined but is associated with autophagosome formation and depends on autophagy proteins, although it is controversial whether cells truly die *via* autophagy, particularly as there are no distinct markers of the process (25). Autophagy may also contribute to oncogene-induced senescence, demonstrated by depletion of ATG5 by shRNA which inhibits oncogene-induced senescence in human fibroblasts (26). There is a growing body of evidence showing that defects in autophagic machinery prevent malignant cell proliferation, for example, metastatic carcinoma cell lines where Beclin-1 or ATG5 is downregulated are unable to survive (27), and siRNA depletion of the essential autophagy gene ATG7 enhances apoptosis in colon cancer cells (28).

In contrast, autophagy may allow established tumors to survive and progress by reducing their sensitivity to stress and cell death signals. Enhanced autophagic response in advanced human tumors correlated with an invasive phenotype and poor prognosis (29). Autophagy also supports tumor cell survival by increasing ATP levels during hypoxia, nutrient deprivation, and detachment from the extracellular matrix, all of which may occur in tumors and would usually result in cell death (9). A number of anticancer therapies have been shown to induce autophagy in human cancer cell lines (30), which may cause cells to become resistant to the therapy, and autophagy inhibition can re-sensitize previously resistant cells to therapy (31).

In summary, the role of autophagy in cancer appears to change during tumor progression. Autophagy protects healthy cells from malignant transformation by maintaining cellular homeostasis and normal metabolism, but after malignant transformation, when presumably autophagy has been suppressed, restoration of autophagy promotes tumor progression, invasion, and metastasis (9). The pro-survival role of autophagy in tumors has been explored as a potential therapeutic target in a number of cell-based studies and clinical trials.

## Molecular Control of Autophagy in Pancreatic Cancer and Its Development

Autophagy is crucial for maintaining cellular homeostasis and has a dual role in cancer as discussed above. It is therefore important to understand autophagy regulation as a degradation and stress-control pathway. The TOR and RAS–cAMP–PKA signaling cascades negatively regulate autophagy and sense nutrient deprivation (which activates autophagy), although details on how multiple signaling mechanisms coordinate in order to control autophagy are not fully understood (32) (Figure 1). Molecular control of autophagy has been widely studied, and the process is tightly regulated at various levels (10).

One of the levels of autophagy regulation is transcriptional control. Transcription of LC3 is upregulated during starvation in mammalian cells (10), a process dependent on the FoxO3 transcription factor (33). Epigenetic changes have also been shown to regulate autophagy, such as the hyperacetylation of histones through histone deacetylase inhibitor treatment, which activates autophagy (34). Posttranslational modification of the autophagy machinery includes phosphorylation of Beclin-1 in response to autophagic stimuli, which is required for maximal autophagy (35).

Despite the accumulation of information on molecular control of autophagy, evidence is just emerging showing these mechanisms (transcriptional, epigenetic, or posttranslational) controlling autophagy are active in PDAC. The evidence on autophagy control in PDAC will be summarized for the purpose of this review.

### Molecular Control of Autophagy in PDAC Survival

A major route in the development of PDAC is through acinar cell damage and dysfunction. Pancreatic acinar cells produce and secrete digestive enzymes and proteases, which require a very high protein biosynthetic rate and an extensive rough endoplasmic reticulum network. Consequently, acinar cells are prone to accumulation of misfolded proteins and ER stress (36, 37). The latter can be involved in the pathogenesis of pancreatitis, which in turn causes inflammation of the exocrine pancreas that may lead to development of PDAC (38). This is particularly likely in the case of chronic pancreatitis (39).

Autophagy is required for the maintenance of acinar cell physiology, as demonstrated by *in vivo* loss of ATG7 in pancreatic epithelial cells leading to pronounced acinar cell damage and loss followed by chronic pancreatitis (16). Primary acinar cells depleted of ATG7 displayed an impaired autophagic canonical flux as LC3-I and p62 protein levels were elevated. Impaired autophagy can lead to an increase in misfolded proteins that undergo ubiquitination and are bound by p62, leading to ER stress and mitochondrial damage (40). Conditional ATG7 knockout mice, in which ATG7 was lacking in all pancreatic epithelial cells, displayed an increase in damaged mitochondria and ER stress, resulting in accumulation of ROS in the pancreata. To counteract these disruptive processes, ATG7-depleted primary acinar cells and pancreata upregulate the transcription nuclear factor erythroid 2-related factor 2 (NRF2), which can stimulate an antioxidative response (16, 41).

A way in which autophagy in acinar cells may be controlled is through VMP1, which triggers the formation of LC3 positive vacuoles when stably expressed in the pancreatic acinar cells of transgenic mice (12). Cell starvation and mTORC1 inhibition induce VMP1 expression (12), and VMP1 is thought to function through interaction with Beclin-1 and recruitment of the PtdIns3P-kinase complex at the phagophore (42). VMP1 transiently localizes with early autophagic structures on the ER membrane (13) and co-localizes with ULK1 at early autophagic structures (13). Furthermore, RNAi experiments in PANC1 cells show that oncogenic KRAS requires VMP1 to induce autophagy. In PDAC cells, VMP1 is upregulated *via* a KRAS–PI3K–AKT1–GLI3-p300 pathway (43). This is of particular interest given that VMP1 was originally identified in rats as a pancreatitis-induced protein restricted to acinar cells (44), so expression of this protein is likely to be an autophagy regulator in PDAC.

As discussed above, accumulation of misfolded proteins and ER stress can be involved in pathogenesis of pancreatitis. ROS generation, which has been shown to regulate autophagy, might contribute to this process (45). In PDAC cell lines, ROS inhibition with an antioxidant significantly reduced basal autophagy levels and, conversely, autophagy inhibition resulted in an increase in ROS levels, confirming a cross-regulation of ROS and autophagy in PDAC (14). The role of ROS and autophagy in PDAC may be biphasic—during early stages of cancer low autophagy levels allow ROS to promote pro-tumorigenic genomic instability required for transformation, whereas in more progressed PDAC cells, autophagy protects the cells from cytotoxic ROS accumulation (14). This has been demonstrated by an increase in markers of double-strand breaks such as 53BP1 foci in PDAC cells where autophagy is inhibited, and this DNA damage in PDAC is thought to allow increasing tumor growth (14).

### Molecular Control of Autophagy in PDAC Development

There is a range of evidence showing that PDAC tumors have constitutively activated autophagy and are dependent on autophagy for survival and development. Measurement of LC3 puncta and LC3-II levels in PDAC cell lines shows elevated basal autophagy levels compared to non-cancerous pancreatic cells and other cancer cell lines (14). Immunohistochemistry analysis of samples from a range of human pancreatic tumors has shown an increase in autophagy levels during the progression from premalignant pancreatic intraepithelial neoplasms (PanINs) to more advanced PDAC (14). The role of autophagy in PDAC progression was probed further by the use of the chloroquine (CQ), which raises the lysosomal pH and thereby inhibits autophagy, to treat mice with advanced PanIN or PDAC, which suppressed tumor growth *in vivo* (14).

However, in a separate mouse study, it has been shown that autophagy deficiency increases PanIN development and tumor initiation, although it makes PanIN progression to PDAC less likely (46). This is supported by evidence showing that autophagy-deficient *ATG7^−/−^* mice show enhanced RAS-driven PanIN formation but do not develop PDAC (20).

The functions of the RAS oncogene and TP53 tumor suppressor in tumorigenesis have been described in detail elsewhere, and aberrations of both of these proteins appear to be cooperative in their contribution to malignancy (47). As well as the high rate of *KRAS* activating mutations, sequence analysis has shown that PDACs demonstrate a mixture of tumor suppressor gene mutations, with TP53 being mutated or inactivated in 75% of PDAC and mutant TP53 being shown to drive pancreatic cancer (48). Autophagy inhibition by CQ treatment or RNAi has been shown to inhibit growth of PDAC cell lines harboring *TP53* mutations. Furthermore, patient-derived xenografts with *TP53* mutations grow slower after autophagy inhibition (46).

In contrast, TP53 status has been shown to determine the role of autophagy in tumor development in mice KRAS mutant pancreatic tumors, where PDAC formation is accelerated by autophagy inhibition in cases where TP53 is absent (20). This may be because TP53-deficient tumors and cell lines have lower numbers of autophagosomes, so their viability is not dependent on the process (20). This indicates that autophagy is not always critical to PDAC tumor development.

Another study indicates that autophagy is dispensable for growth of KRAS mutant tumors and cell lines (19). Forty-seven human cancer cell lines were treated with the CQ derivative Lys01 or shRNA to remove autophagic machinery components such as ATG7, revealing that KRAS-mutated cells are no more dependent on autophagy than their wild-type counterparts (19). This was supported by *in vivo* experiments where autophagy inhibition did not reduce growth of a KRAS mutant tumor derived from the PDAC cell line Panc10.05 (19). These findings raise questions regarding the assumption that inhibition of autophagy reduces cell growth and viability of KRAS mutant PDAC cells and could mean that the function of autophagy is to support tumor growth through host tissues, such as cancer-associated fibroblasts (49).

### Hypoxia-Induced Autophagy in PDAC

Preexisting vasculature of normal tissue has been shown to be insufficient to support the requirements of tumors for nutrients and oxygen, and in particular, the pancreatic tumor microenvironment has been found to be hypoxic (50). Higher tumor levels of hypoxia as measured by hypoxia-inducible factor 1α (HIF-1α) expression have been shown to correlate with poor prognosis in patients with PDAC (51, 52).

The cellular response to hypoxia may contribute to elevated basal autophagy levels in PDAC, as autophagy can be induced by hypoxia in several ways. First, HIF-1α has been shown to upregulate Bcl-2/adenovirus E1B 19-kDa protein-interacting protein 3 (BNIP3) and BNIP3 like protein (BNIP3L). BNIP3 and BNIP3L subsequently disrupt the Bcl-2–Beclin-1 complex in an mTOR-independent way, which induces autophagy (53). This mechanism has been demonstrated in various cancer cell lines, including prostate cancer and salivary adenoid cystic carcinoma, and we speculate that this process may occur in PDAC (54, 55). In contrast, another study suggests hypoxia-induced autophagy in tumor cells is dependent on AMP-activated protein kinase and mTOR, thus excluding a role for HIF-1α, BNIP3, and BNIP3L (56). In a hypoxic tumor microenvironment, the unfolded protein response can facilitate autophagy. This mechanism involves the PKR-like endoplasmic reticulum kinase-activating transcription factor 4 (ATF4) pathway. ATF4 is able to bind a cyclic AMP response element binding site in the LC3B promoter inducing LC3B transcription (57). This ATF4-mediated transcriptional LC3B induction results in replenishment of LC3B levels during extended periods of hypoxia characterized by high autophagic flux (58).

Evidence for the connection between autophagy and hypoxia in PDAC tumors is high levels of LC3, which has been shown to be associated with the hypoxic marker carbonic anhydrase IX at the peripheral area of the pancreatic cancer tissue (59). Under intermittent hypoxia, pancreatic cancer cells demonstrated enhanced invasive ability and increased levels of the cancer stem cells (CSC) marker CD133. In these cells, enhanced autophagy was correlated with elevated HIF-1α levels. The metastatic ability and epithelial-to-mesenchymal transition of pancreatic CSC was also associated with HIF-1α and autophagy (60). These findings are consistent with a previous report showing autophagy to increase survival and migration of pancreatic tumor-initiating CSCs under hypoxic conditions (61). Recent research in the pathways underlying hypoxia in PDAC revealed that hypoxia induces ROS production which subsequently inhibits the pAKT/mTORC1 pathway, inducing autophagy. This process results in a decrease in MUC4 protein levels (an oncogenic transmembrane protein expressed during the early preneoplastic stage). MUC4 degradation decreases growth and survival, potentially providing other stressed cells with required metabolites (62).

In conclusion, although there is significant evidence linking hypoxia and autophagy in PDAC and the translational relevance of this connection, the precise mechanism for hypoxia-induced autophagy in PDAC is not fully elucidated.

## Molecular Pathways Involved in Autophagy and Its Impact on Pancreatic Cancer Metabolism

Autophagy plays a major role in PDAC metabolism, although not all pathways involved in activating and reprogramming autophagy in this context are fully elucidated. Autophagy in PDAC can be seen as part of a broader transcriptional program that coordinates lysosome function and nutrient sensing by the MiT/TFE subclass of basic helix–loop–helix transcription factors including TFE3, MITF, and TFEB, ensuring sufficient levels of intracellular amino acids (11). PDAC cells display an increased lysosomal biogenesis accompanying their expanded autophagosome compartment. In normal cells under nutrient stress, biogenesis of autophagy–lysosome proteins is under control of the MiT/TFE transcription factors (63). RNAseq data across 10 tumor types revealed a high relative expression of these transcription factors in PDAC (11). MiT/TFE proteins act selectively in PDAC cells to regulate a broad autophagy–lysosome program under basal conditions. Despite displaying intact mTORC1 signaling, which phosphorylates MiT/TFE proteins in fed conditions in non-PDAC cells and ensures their cytoplasmic retention (64), PDAC cells show constitutive nuclear localization of each MiT/TFE protein. The cytoplasmic retention mechanism of MiT/TFE in PDAC cells is overwritten by importin-8 (IPO8), a member of the importin-β family of nucleocytoplasmic transporters (65). In PDAC cells, in contrast to non-PDAC cells, IPO8 binds TFE3 resulting in its nuclear translocation and upregulation of its transcriptional program regardless of the nutritional condition (11). Endogenous binding of IPO8 to MITF or TFEB was not shown; however, a combinational depletion of IPO8 and its homolog IPO7 in PDAC cells decreased MITF and TFEB protein levels.

Depletion of MiT/TFE proteins across several PDAC cell lines revealed a regulatory role for MiT/TFE proteins in autophagic flux and lysosomal catabolism. This enables efficient processing of cargo from autophagy and macropinocytosis. Thus, the MiT/TFE protein system provides PDAC cells with both intracellular and extracellular nutrient supplies (11). In nutrient-depleted conditions, PDAC cells rely on the autophagy–lysosome system to maintain intracellular amino acid pools. *In vitro*, silencing of MiT/TFE proteins impaired growth of PDAC cells. TFE3 and MITF were also required for *in vivo* xenograft growth of several PDAC cell lines (11).

**Figure 2:**
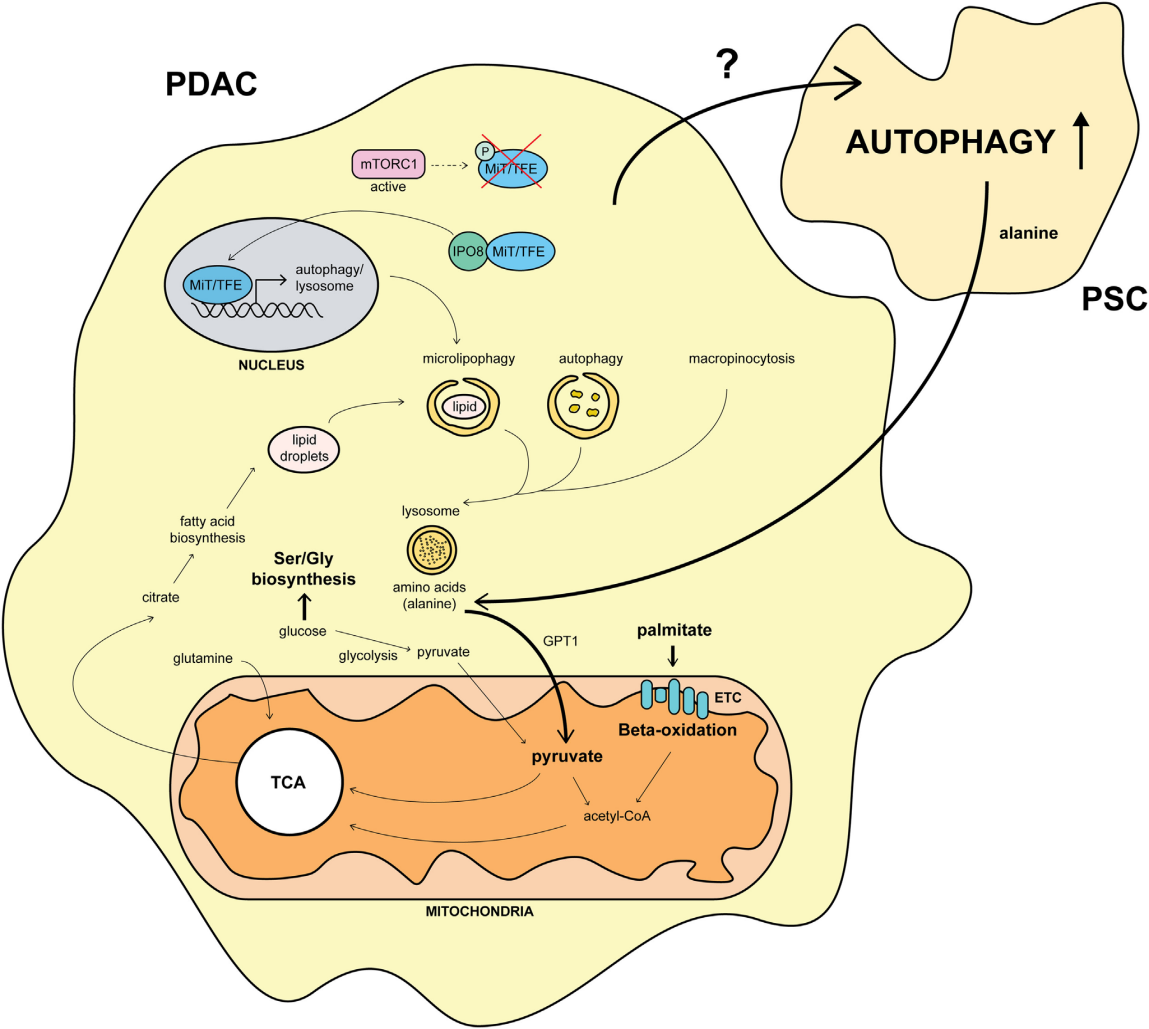
**Overview of the molecular pathways involved in autophagy and its impact on pancreatic cancer metabolism**. Constitutive nuclear import of MiT/TFE proteins by importin-8 (IPO8) upregulates the autophagic-lysosomal machinery in pancreatic ductal adenocarcinoma (PDAC) (11). Autophagy fuels the TCA cycle by amino acid production, shifting the usage of glucose from glycolysis to Ser/Gly biosynthesis (67). This mechanism is further strengthened by pancreatic stellate cells (PSCs) secreting alanine upon autophagy stimulation by unknown PDAC factors (15). Furthermore, the necessity for palmitate feeding oxidative phosphorylation is upregulated in PDAC. Citrate, produced during the TCA cycle, is used for fatty acid biosynthesis, leading to lipid droplet formation and microlipophagy (67). This process, together with the macropinocytotic uptake of extracellular proteins, amplifies the autophagic system in PDAC (11, 67). Arrows and names in bold mark an upregulated pathway in PDAC.

In summary, overriding MiT/TFE inactivation by mTORC1 *via* IPO8-driven nuclear import enables PDAC cells to maintain their intracellular amino acid pool by activation of both autophagy and lysosomal catabolism (Figure 2) (11). This system resembles the constitutive nuclear import of the pro-oncogenic protein eIF4E (a downstream target of mTORC1) in acute myeloid leukemia patients by IPO8 (66) and might be a general mechanism used by several cancer types.

In addition to MiT/TFE-regulated autophagic-lysosomal catabolism, PDACs are also dependent on mitochondrial oxidative phosphorylation for their energy supply. Using an inducible mouse model of mutated KRAS in a *TP53* heterozygous background, Viale et al. showed that repression of mutant KRAS resulted in regressed growth of implanted cells isolated from primary tumors, followed by a relapse after 4–5 months (67). This suggests that a fraction of dormant tumor cells survive oncogene ablation [surviving cells (SCs)]. SCs may possess CSC characteristics as only CD133^+^ CD44^high^ cells were able to avoid apoptosis (67–69). Transcriptomics analysis showed genes involved in the mitochondrial electron transport chain (ETC), lysosome activity, and autophagy are upregulated in SCs. A hyperactive ETC and increased ROS production are hallmarks of SCs. SCs operate close to their maximum respiratory chain capacity and fail to increase glycolysis upon oxidative phosphorylation inhibition in a manner sufficient to maintain ATP production. Furthermore, SCs seem to rely more on pyruvate and palmitate than glucose and glutamine to generate TCA intermediates (67). This is consistent with previous data reporting activation of anabolic glucose and glutamine metabolism in PDAC by oncogenic KRAS (70, 71).

The dependence on oxidative phosphorylation by SCs for their survival was demonstrated by oligomycin treatment of a tumor regression mouse model. Tumors were grown in a mutant *KRAS/TP53* heterozygous-inducible mouse model and regressed upon doxycycline withdrawal. When reintroducing mutant KRAS, 25% of oligomycin-treated mice survived longer than 60 days while vehicle-treated mice survived on average 15 days (67). Mitochondrial respiration would thus make an attractive druggable target to eradicate SCs in PDAC. The ETC dependence of SCs is consistent with previous reports showing both normal and leukemic stem cells rely on mitochondrial respiration (Figure 2) (72).

The role of autophagy in pancreatic cancer metabolism is not restricted to just PDAC cells, but cells surrounding tumors also use autophagy for their energy supply. The surrounding environment heavily influences PDAC metabolism, for example, the stroma enveloping PDAC cells impairs vascularization of tumors leading to a hypoxic, nutrient-poor environment (73). Recently, a new role has been described for stroma-associated PSCs in governing PDAC metabolism. When treating PDAC cells with conditioned medium from a human PSC cell line, the oxygen consumption ratio in PDAC cells increased independent of the presence of serum (15). This effect was attributed to alanine secreted by PSCs. The increase in intracellular alanine concentrations in PDAC cells by PSC-derived alanine could even be further induced by silencing of GPT1, the alanine transaminase responsible for transamination of alanine to form pyruvate and glutamate. Alanine-derived pyruvate did not contribute to glycolytic intermediates but was used in mitochondria as a major source for the TCA cycle as citrate was the main recipient of carbon originated from alanine (15). This alanine-derived carbon would then further fuel fatty acid biosynthesis and could supplant glucose-derived carbon in TCA cycle metabolism, enabling glucose to be used for additional biosynthetic functions (for example, serine/glycine biosynthesis) (15, 71).

Surprisingly, treatment of PSCs with PDAC-conditioned medium significantly increased autophagic flux in PSCs and depletion of ATG5 and ATG7 in PSCs abolished alanine secretion. These findings reveal a two-way intra-tumor metabolic crosstalk in which PDAC signals to PSCs resulting in autophagy induction in the latter, followed by PSC-derived alanine secretion which can fuel the TCA cycle in PDAC. This process is of significant importance under low-nutrient conditions, which mimic the nutrient-deprived PDAC environment. *In vivo*, co-injection of PDAC cells with autophagy-impaired PSCs decreased tumor growth and kinetics, also in orthotopic assays.

An unresolved question in the PDAC–PSC crosstalk is how PDAC stimulates autophagic flux in PSCs (Figure 2). Just as autophagy inhibitors in PSCs may be effective, this could be a new avenue for therapeutic intervention, restraining PDAC growth and sensitizing tumors to chemotherapy. One possible mechanism may be regulated by TGF-β1 secretion from PDAC. Activation of PSCs by TGF-β1 transforms them to an activated myofibroblast-like phenotype where synthesis of excessive amount of extracellular matrix proteins causes fibrous tissue formation (74). Furthermore, TGF-β1 was shown to induce autophagy in hepatic stellate cells (75) so a similar mechanism may be at play in PSCs although other cytokines secreted by PDAC cells could also be involved in this process.

Future therapies targeting the metabolism of PDAC are likely to be directed toward fighting a multifront battle. The autophagic component is one important aspect of this battle but will need to be part of a combined approach to drain or disrupt PDAC energy supplies.

## Potential for PDAC Therapy through Autophagy Modulation

Autophagy inhibition is a promising avenue for therapeutic treatment of PDAC. One of the first clinical trials aimed at inhibiting autophagy in PDAC used hydroxychloroquine (HCQ), which did not demonstrate a significant therapeutic effect as a monotherapy (17). However, the HCQ doses tested in this study may have been inadequate to consistently inhibit autophagy, and patients tested were suffering from previously treated metastatic tumors. It should be noted at this point that the antiproliferative effects of CQ were shown to be autophagy independent as both ATG7-deficient and -proficient cells were equally sensitive to CQ (19). This implies that data from clinical trials involving CQ as an autophagy inhibitor should be interpreted with caution. More promising were results from Yang et al. who showed CSCs *in vivo* were more susceptible to gemcitabine treatment upon autophagy inhibition, and combined treatment was more effective than either agent alone in preventing pancreatic tumor formation (18). In addition, as Viale et al. proved in SCs that ETC, lipophagy, and autophagy are all critical for the survival of SCs (67), inhibition of autophagy alone still leaves alternative pathways for PDAC energy production. Thus, the potential efficacy of a monotherapy inhibiting autophagy in PDAC is low, and combinational therapies are preferential. An overview of autophagy-related PDAC therapies currently being tested in clinical trials listed on the US website http://Clinicaltrials.gov can be found in Table 1.

**Table 1:** **Overview of autophagy-related pancreatic ductal adenocarcinoma (PDAC) therapies currently being tested in clinical trials listed on the US website http://Clinicaltrials.gov**.

Aim	Agent	Trial design	NCT number
Autophagy inhibition in PDAC and assessment of JNK1 as PDAC biomarker	Hydroxychloroquine (HCQ) Gemcitabine	Phase I/II	NCT01506973
Determine the ability of HCQ to improve a pre-operative regime of gemcitabine/nab-paclitaxel in patients with potential resectable PDAC	HCQ Gemcitabine Abraxane	Randomized phase II	NCT01978184
Test efficacy of HCQ/gemcitabine combined treatment in PDAC patients before surgery	HCQ Gemcitabine	Phase I/II	NCT01128296
Determine whether a combinational therapy of HCQ/radio therapy/capecitabine can control tumor growth	HCQ Capecitabine Proton/photon radio therapy	Phase II	NCT01494155

Investigation is ongoing into a number of possibilities for combinational PDAC treatment involving autophagy inhibition. MAPK and NF-κB inhibition could be a promising strategy. PANC1 and MIA-PaCa-2 PDAC cell lines were treated with U0126 (a MAPK inhibitor) or caffeic acid phenethyl ester (CAPE, an NF-κB inhibitor), producing a strong inhibition of tumor cell growth without inducing apoptosis. Autophagy inhibition by (3-MA, an inhibitor of PI3K, which blocks autophagosome formation) followed by PDAC treatment with U0126 or CAPE caused a significant apoptotic response (76). A combinational treatment including MAPK/NF-κB/autophagy inhibitors might thus be an interesting avenue.

More evidence supporting a combinational therapy involving NF-κB inhibitors was provided by Yang et al. (18). This study also emphasizes the need for reliable prognostic markers for PDAC. The high metastatic potential and resistance to chemotherapy and radiation therapy in several cancers have been linked to CSCs (77–79). Presence of CSCs is associated with poor outcome for patients diagnosed with pancreatic cancer (80). A putative marker for CSCs, other than the aforementioned CD133 and CD44, is aldehyde hydrogenase 1 (ALDH1) (81). Another marker associated with poor prognostic outcome in several cancers is osteopontin (OPN), a secreted glycoprotein able to interact with CD44 and activate several downstream signaling pathways such as growth factor receptor signaling *via* PI3K/AKT, NF-κB, and MEK/ERK (82–85). High expression of LC3 combined with high levels of ALDH1 is associated with shorter overall survival and disease-free survival in pancreatic cancers patients, making coexpression of LC3/ALDH1 a valuable prognostic PDAC marker (18). Autophagy inhibition by silencing of ATG5, ATG7, or Beclin-1 *in vivo* rendered tumors markedly more susceptible to gemcitabine treatment. A combined treatment of CQ and gemcitabine was more effective than either agent alone in preventing pancreatic tumor formation *in vivo* (18). Autophagy blockade boosted the susceptibility of pancreatic CSCs to gemcitabine and thus enhanced the efficacy of gemcitabine against pancreatic cancer. OPN was found to upregulate CSC activity by activating autophagy. OPN can exert its functions by triggering the NF-κB, MEK/ERK, and p38 MAPK in PDAC cells. Pretreatment with BAY 1170–82, an NF-κB inhibitor, could effectively block the OPN-mediated LC3-II increase in PANC1 cells (18). A role for OPN is also found in breast cancer, where its expression associates with cancer aggressiveness. Depletion of OPN in breast cancer cells inhibited the class I PI3K/AKT/mTOR pathway, promoted expression of LC3 and Beclin-1, and increased apoptosis (86). Pharmacological autophagy and NF-κB inhibition have not been tested in this context.

KRAS was considered another interesting prognostic marker for PDAC. As oncogenic KRAS has been described as a contributing factor in PDAC addiction to autophagy, it was suggested that the mutation status of RAS could identify patients who would be more susceptible for HCQ treatment (87). This biomarker avenue turned out not to be beneficial for patient selection as oncogenic KRAS did not always promote autophagy (88). As mentioned above, oncogenic KRAS can have both stimulating and repressive effects on autophagy, and these differing effects are tumor cell-specific and context-dependent. Considering CQ sensitivity, KRAS activation rendered some cell lines more susceptible to CQ while others became more resistant (88). This is in line with the findings of Rosenfeldt et al. who suggest that in the absence of TP53, autophagy is no longer required for KRAS-mediated tumor development in PDAC, although this study does not indicate that p53 status predicts the response to anti-autophagic therapy for a developed tumor (20). Thus, the quest for suitable biomarkers identifying PDAC patients susceptible to autophagy inhibition is currently still ongoing.

## Conclusion and Future Directions

Autophagy has roles both in protection from malignant transformation and in promotion of tumor progression and survival. In the case of PDAC, a significant body of evidence points to a pro-tumorigenic autophagy role, where the constitutive activation of this process allows cell survival and promotes metabolism.

The mechanisms for this are diverse and require consideration of both the tumor itself and the surrounding tissue, such as stroma-associated PSCs, which provide metabolic support for the tumor by secreting alanine through cancer cell-stimulated autophagy, hence fueling the TCA cycle, Ser/Gly biosynthesis, and fatty acid synthesis in PDAC cells (15). Within the PDAC cells, it is thought that autophagy is constitutively active and is regulated through transcriptional control (11) and ROS-related signaling (45). Surrounded by a stressful environment, therefore, one way PDAC cells can upregulate their energy production components to fuel their expansion and migration is through autophagy. Autophagic genes and flux are upregulated in PDAC, as are the lysosomal and oxidative phosphorylation systems (67). MiT/TFE proteins play a crucial role in the basal transcriptional upregulation of autophagy in PDAC (11). Furthermore, upregulated autophagy is important for survival of these cells, as demonstrated by studies where autophagy is either pharmacologically or genetically impaired, resulting in loss of viability in PDAC cell lines and pancreatic cancer xenograft regression (14). PDAC progression has also been shown to rely on autophagy, although this appears to be dependent on TP53 status. In cases where TP53 is absent, tumors and cell lines are actually accelerated by autophagy inhibition (20), highlighting the need for biomarkers to report autophagy inhibition in PDAC.

Pancreatic cancer is a cancer of unmet need (89). The requirement of many pancreatic cancers for constitutively activated autophagy makes targeting this pathway an attractive new therapeutic avenue. However, due to the various feedback loops, crosstalk and parallel energy supply systems in PDAC, it might be challenging to impair PDACs’ energy metabolism by autophagy inhibition on its own. Early clinical trials have shown that autophagy inhibition as a monotherapy may not be sufficient (17), but clinical trials involving combination treatment of an autophagy inhibitor and chemotherapy treatments are ongoing. In this light, the development of new, more effective upstream autophagy inhibitors of autophagy also has great potential.

## Author Contributions

MN and TVA wrote most of the manuscript and made the figures. JL, JS, and KR thoroughly revised and amended the manuscript. ST conceived, thoroughly revised, and amended the manuscript.

## Conflict of Interest Statement

The authors declare that the research was conducted in the absence of any commercial or financial relationships that could be construed as a potential conflict of interest.
